# Association between the content of intracellular and extracellular fluid and the amount of water intake among Chinese college students

**DOI:** 10.1186/s12986-019-0397-9

**Published:** 2019-09-18

**Authors:** Na Zhang, Jianfen Zhang, Songming Du, Hairong He, Xinyu Yan, Guansheng Ma

**Affiliations:** 1 Department of Nutrition and Food Hygiene, School of Public Health, Peking University, 38 Xue Yuan Road, Hai Dian District, Beijing, 100191 China; 2 Laboratory of Toxicological Research and Risk Assessment for Food Safety, Peking University, 38 Xue Yuan Road, Hai Dian District, Beijing, 100191 China; 3 Chinese Nutrition Society, Room 1405, Broadcasting Mansion, 14 Jianguo Wai Street, Chaoyang District, Beijing, 100029 China

**Keywords:** Intracellular fluid, Extracellular fluid, Total body water, Total fluid intake, Water intake from food

## Abstract

**Background:**

Normal distribution of body fluid is important for maintaining health through the balance of water metabolism. Studies have shown that disease states and diuretics perturb the balance and then induce abnormal intracellular/extracellular fluid ratio. However, there are relatively few researches on the associations between water intakes and body fluid. The objective of this study was to explore the association between body fluid and water intake.

**Methods:**

A total of 159 young adults in Baoding, China were recruited in this cross-sectional survey and completeness of follow-up was 98.1%. A 7-day fluid specific diary was used to record total fluid intake (TFI). Water intake from foods (FWI) for 3 days was measured using the methods of weighting, duplicate portion method and laboratory analysis by researchers. Body fluid was measured using bioelectrical impedance analysis.

**Results:**

Total body water (TBW), intracellular fluid (ICF) and extracellular fluid (ECF) of participants were 32.8[28.0,39.2], 20.5[17.3,24.5] and 12.4[10.7,14.7], (kg). This represented 55.2 ± 6.2, 34.4 ± 4.0 and 20.8 ± 2.3 (%) of body weight (BW), respectively. ICF, ECF and TBW among male participants who drank more than or equal to adequate TFI was higher than those who drank less (*Z* = -1.985, *p* = 0.047; *Z* = -2.134, *p* = 0.033; *Z* = -2.053, *p* = 0.040). Among both males and females, the values of TBW/BW in participants whose TWI met or exceeded the AI were higher than those with TWI less than AI (*t* = − 2.011, *p* = 0.046; *t* = − 2.716, *p* = 0.008). Among all participants, there was moderate correlation between water intakes (TFI/BW, FWI/BW and TWI/BW) and body fluid (ICF/BW,ECF/BW and TBW/BW) (*p* < 0.01 for all). Same correlations were found among both males and females.

**Conclusion:**

There is a certain degree of association between water intake and body fluid. However, whether TFI or TWI achieve AI or not do not disturb the balance on the distribution of body fluid. More studies should be conducted to find the diagnostic threshold on TFI and TWI which may disrupt the distribution of body fluid so as to prevent related diseases.

**Trial registration:**

Chinese clinical trial registry. Name of the registry: Relationship of drinking water and urination. Trial registration number: ChiCTR-ROC-17010320. Date of registration: 01/04/2017. URL of trial registry record: http://www.chictr.org.cn/edit.aspx?pid=17601&htm=4.

## Background

Water is essential and necessary for development and survival [1]. Water involve in the metabolism of body, modulate normal osmotic pressure, maintain electrolyte balance and regulate body temperature. Water is the largest constituent of body tissues. For babies within 0~6 months, total body water accounts for about 80% of body weight. The percentage gradually reduces with the growth of age. For adults, total body water accounts for about 60~70% of total body weight [1]. For elders, the percentage gradually reduces as the reduction of muscle tissues. The percentage in female is less than that in male [1–3]. The body obtains water from drinking fluids, water in food and metabolic water [4]. Water is lost from the body as urine, sweat, exhaled breath, and in feces [5]. Sources and losses of water are in dynamic balance and are maintained at approximately 2500 mL among adults [1]. The inner balance of water metabolism mainly depends on the body’s own homeostatic regulatory system, which is regulated by the thirst center in the brain, vasopressin secreted by the posterior pituitary gland and the kidneys [1, 6, 7]. The thirst center is an important link in regulating the source of water in the body [8–11]. The kidney regulates water balance by urination, dilution and concentration of urine. Under normal circumstance, the body fluid is maintained in a dynamic balance, by osmotic pressure balance, through the osmotic action of solutes in the intracellular fluid (ICF) and extracellular fluid (ECF). The osmotic pressure balance of ICF and ECF mainly depends on the simple diffusion of water molecules inside and outside the cell. The balance between anionic and cationic electrolytes of ICF and ECF is regulated through exchange of these electrolytes [2]. When water intake is insufficient or water loss is excessive, the osmotic pressure of ECF increases. Drinking behavior is stimulated aroused through the regulatory mechanism of nervous system [12]. In addition, the secretion of antidiuretic hormone and aldosterone increase, which changes water permeability of the distal renal tubules and collecting tubules of the kidney to increase water reabsorption, reduce water discharge, and maintain normal osmotic pressure of body fluid [9, 13, 14]. On the contrary, if too much water is taken, the secretion of these hormones are inhibited, resulting in increased urine output [1]. Therefore, it is probable that fluid intake will influence the distribution of body fluid.

However, currently few studies have looked the relationship of water intake on the distribution of body fluid. Such studies have mainly focused on disease states including hepatitis, hepatocirrhosis, and kidney disease requiring dialysis [15, 16].

Studies on water intake focus on the amount and the type of fluid intake, behaviors related to water intake, water intake and hydration, and water intake and health. However, the importance of water and hydration does not have enough attention. More related studies are needed to be initiated. In China, even the studies on water intake were few. Among these scanty studies, it was found that approximately 32% of the adults drank less fluid than the recommended amount on daily total fluid intake (TFI) set by the Chinese Nutrition Society in 2007 (1200 mL/day) [17]. Nearly two-thirds of the participants drank less than the recommended amount on TFI [18]. And 25% of young male adults achieved the adequate water intake for TFI set by the Chinese Nutrition Society in 2013 [1, 19]. However, no research has measured body fluid and water intake simultaneously in healthy participants.

Unbalanced shift in body fluid are not only effective indicators at diagnosing dehydration and water intoxication [20], but also risk indicators for some diseases, such as cardiovascular disease in hemodialysis patients, diabetic ketoacidosis, kidney diseases, and so on [21, 22]. The maintenance of the normal ECF/ICF ratio is important for heath. Body fluid is regulated through the balance of water metabolism, and the balance of water metabolism is closely related to the sources and loss of water [1]. Studies have shown that disease states and diuretics perturb this balance but we do not know how water intake affects it [23, 24]. It is necessary to explore the association between water intake and body fluid based on more related study.

Young adults with similar age and lifestyle were recruited from a university in this study to maximize the exclusion of disease and other confounding factors. The aims of present study were, firstly, to evaluate the water intake of participants; secondly, to analyze the relationship between water intake and body fluid.

## Materials and methods

### Study design

This is a cross-sectional survey on fluid intake and body water which lasted for 7 days.

### Study participants

A simple random sampling method was used to select participants from a university in Baoding, Hebei Province, China.

Inclusion criteria were aged between 18 and 23 years.

Exclusion criteria were smoking, habitual high alcohol consumption (drinking alcohol > 20 g/day); type 2 diabetes mellitus, gastrointestinal tract disease, oral cancer, cleft palate, kidney disease or other chronic diseases and neuroendocrine disorders.

### Sample size calculation

The formula $n={\left(\frac{Z_{1-\alpha /2}\sigma }{\delta}\right)}^2$ was used to calculate the sample size for survey on water intake and body fluid. It was reported that the daily total fluid intake of adults was 1342 mL, standard deviation σ was 653 mL and tolerance error *δ* was 100 mL based on the reference of previous literature [19]. And the total body water of Asia adults was 35 L, standard deviation σ was 8 L and tolerance error *δ* was 1 L based on the reference of previous literature [25]. Then, software R 3.6.1 (MathSoft, Inc., St. Louis, Missouri, USA) was used to calculate the sample size for the association between water intake and body fluid. The programming in R 3.6.1 to calculate sample size was “pwr.r.test (r=0.3, sig.level = 0.95, power = 0.80, alternative = “greater”)”. Statistical significance *α* was set at 0.05 (*p* < 0.05, 2-tailed) with 95% confidence. In addition, 10% drop-out rate is taken into account. Eventually, 160 participants were needed in this study with half men and half women.

### Ethics

The study protocol and instruments were reviewed and approved by the Peking University Institutional Review Committee. Ethical approval identification code is IRB00001052–16071. The study was conducted according to the guidelines of the Declaration of Helsinki. Prior to the study, all participants read and signed the informed consent form.

### Study design and procedure

**Fig. 1:**
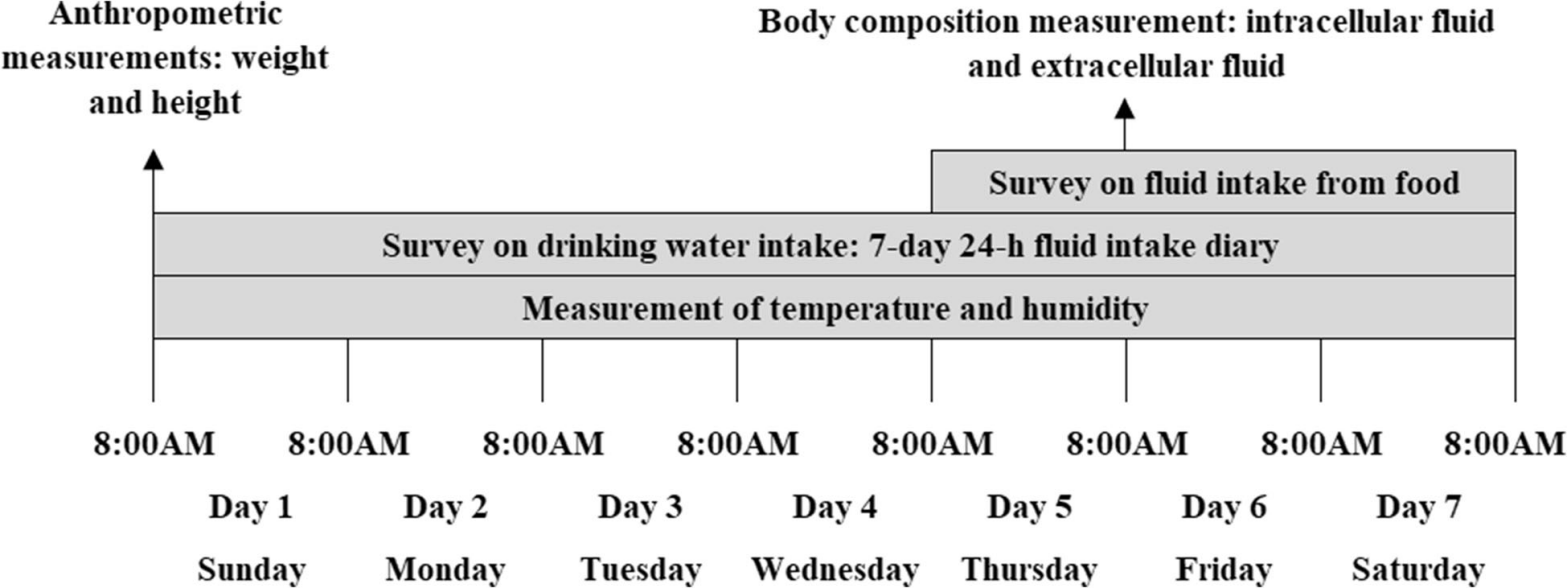
Study procedure

Total 159 participants were recruited in this study. Among them, 156 participants completed this study and completeness of follow-up was 98.1%. A 7 days cross-sectional survey was performed in spring 2017. The experimental site was set in the school where the participants in for their convenience to come to every day for 7 days. During that week, participants finished this study in their free-living environment in school as usual. Their food and drink was ad-libitum. They were told not to do high-intensity physical activities. The study procedure is shown in Fig. 1. On day 1, all participants arrived at the research center for anthropometric measurements at 8:00 AM. Anthropometric measurements of height and weight were conducted following a standardized procedure by trained staff before breakfast. In order to assess daily total fluid intake (TFI), participants were asked to complete a self-administered 7-day 24-h fluid intake diary from day 1 to day 7 after training. On days 5–7, the foods eaten by participants were weighed and the water content of the food was analyzed on three consecutive days (including two weekdays and one weekend day) to assess daily water intake from food (FWI). On day 6, body composition was performed using bioelectrical impedance analyzer following a standardized procedure by trained staff. Temperature and humidity of both indoors and outdoors at the study site in university were recorded at 10:00 AM, 2:00 PM and 8:00 PM every day by researcher.

### Definition

Daily total fluid intake (TFI) included the amount of water (plain water, bottled water, etc.), dairy and dairy products, tea (brewed green tea, fermented or non-fermented tea, etc.), sugar-sweetened beverages (carbonated drinks, protein drinks, functional drinks, fruit juices with added sugar, and other sugar-sweetened beverages), alcoholic drinks (wine, beer, etc.) and other beverages.

Daily water intake from food (FWI) included water intake from staple foods (steamed rice, steamed bun, flatbread, etc.), dishes (cooked vegetables, meat, fish, etc.), porridge (millet congee, rice gruel, etc.), soup and snacks (fruits, nut, etc.)

$$ \mathrm{Daily}\ \mathrm{total}\ \mathrm{water}\ \mathrm{intake}\ \left(\mathrm{TWI}\right)\ \left(\mathrm{mL}\right)=\mathrm{TFI}\ \left(\mathrm{mL}\right)+\mathrm{FWI}\ \left(\mathrm{mL}\right) $$

### Assessment of fluid intake

Total fluid intake (TFI): Data on TFI of the participants was collected using a self-administered, validated 7-day, 24-h fluid intake diary [17, 19, 26]. Participants were instructed on how to use the fluid intake diary. The amount of fluid intake for each time was measured using a cup to the nearest of 5 mL.

Water intake from food (FWI): Participants could eat or drink anything they wanted and in any amount they wanted. The methods of weighting, duplicate portion method and laboratory analysis were used to assess FWI for three consecutive days (including two weekdays and one weekend day), which has been verified valid in previous study [19]. All foods consumed by the participants were prepared in two portions, one for their consumption and the other for backup food samples. All foods consumed by the participants were weighed accurately by trained investigators using electronic balance (YP20001; SPC; Shanghai, China). Backup food samples were stored in refrigerators at 4 C and sent to laboratory to be measured within 36 h. Water in food was measured according to National Food Safety Standard GB 5009.3–2016 *Determination of water in Food* by laboratory analyst in Beijing Institute Nutritional Resources [27]. Parallel samples were taken for each food samples, and the error between the two results was no more than 5%. Water from fruits was assessed by trained investigators using the China food composition table [28].

$$ \mathrm{Food}\ \mathrm{consumed}\ \mathrm{by}\ \mathrm{participants}\ \left(\mathrm{g}\right)=\mathrm{Food}\ \mathrm{before}\ \mathrm{consumption}\ \left(\mathrm{g}\right)\hbox{-} \mathrm{Food}\ \mathrm{after}\ \mathrm{consumption}\ \left(\mathrm{g}\right). $$

$$ \mathrm{Ratio}\ \mathrm{of}\ \mathrm{water}\ \mathrm{content}\ \mathrm{in}\ \mathrm{food}\ \left(\%\right)=\left(\mathrm{Weight}\ \mathrm{of}\ \mathrm{container}\ \mathrm{and}\ \mathrm{food}\ \mathrm{sample}\ \mathrm{before}\ \mathrm{drying}-\mathrm{Weight}\ \mathrm{of}\ \mathrm{container}\ \mathrm{and}\ \mathrm{food}\ \mathrm{sample}\ \mathrm{after}\ \mathrm{drying}\right)/\mathrm{weight}\ \mathrm{of}\ \mathrm{food}\ \mathrm{sample}\times 100\%. $$

$$ \mathrm{FWI}\ \left(\mathrm{mL}\right)=\mathrm{Food}\ \mathrm{consumed}\ \mathrm{by}\ \mathrm{participants}\ \left(\mathrm{g}\right)\times \mathrm{Ratio}\ \mathrm{of}\ \mathrm{water}\ \mathrm{content}\ \mathrm{containing}\ \mathrm{in}\ \mathrm{food}\ \left(\%\right)/1.0\mathrm{g}/\mathrm{mL}. $$

### Anthropometry

Height were measured by trained investigators with a height-weight meter (HDM-300; Huajun, Zhejiang, China) in bare feet. Height was measured twice to the nearest 0.1 cm.

Intracellular fluid (ICF), extracellular fluid (ECF) and body weight (BW) were measured by trained investigators with a bioelectrical impedance analyzer (Inbody 720; Inbody; Seoul, Korea) in fasting state [29–31]. Participants took off their shoes and caps and wore light clothes. Four fingers of participants touched the surface of the hand electrode fixator, and the thumb was gently placed on the electrode of the thumb. During the whole process of examination and analysis, they always held the electrode of the hand gently. Participants stood barefoot on the sole electrode, and the whole sole of foot was in close contact with the foot electrode. The angle between the trunk and the upper limb of participants was kept at about 15 degrees. During the process of examination, participants stood relaxed without flexing their muscles. After the data displayed in the liquid crystal display of the bioelectrical impedance analyzer was stabilized, the investigators read and recorded the results. Before the examination, participants could not do physical activities, eat something or bathe before the examination.

$$ \mathrm{Total}\ \mathrm{body}\ \mathrm{water}\ \left(\mathrm{TBW}\right)\ \left(\mathrm{mL}\right)=\mathrm{ICF}\ \left(\mathrm{mL}\right)+\mathrm{ECF}\left(\mathrm{mL}\right) $$

### Temperature and humidity of the environment

The temperature and humidity both indoors and outdoors at the study site were measured using a temperature hygrometer (WSB-1-H2, Exasace, Zhengzhou, China) by researcher at 10:00 AM, 2:00 PM and 8:00 PM each day during the 7 study days.

### Statistical analyses

A database was built using EpiData3.1 software and data was entered with the double entry method. SAS 9.2 (SAS Institute, Inc., Cary, NC, USA) was used for the statistical analyses. TFI was an average for 7 days, and FWI was an average for 3 days. ICF and ECF were the value for 1 day. Mean and standard deviation (SD) was used to describe variables in normal distribution; percentile (P50 [P25, P75]) was used to describe variables in skewness distribution; and count data were presented as n (percentage). TFI and TWI of participants were compared with the recommendation for the adequate water intake (AI) for TFI and TWI set by *Chinese Nutrition Society (2013).* The differences of variables in normal distribution between sexes were analyzed using Student’s *t*-test; the differences of variables in skewness distribution between sexes were analyzed with the method of *Kruskal Wallis*; and the differences in count data were analyzed using *χ2* test (the index on the percentage of TFI/TWI achieving related AI). *Pearson* and *Spearman* partial correlation coefficient were performed to determine the strength of the associations between water intake and body fluid. Multivariate linear regression model was used to investigate the predicators of studying variables of interest, including ICF/BW, ECF/BW, TBW/BW and ECF/ICF. Significance level was set at 0.05 (*p* < 0.05).

## Results

### Characteristics of the participants

A total of 159 participants was recruited, 156 completed the study with a 98.1% completion rate, including 80 males and 76 females. The characteristics of the participants were shown in Table 1.

**Table 1:** Characteristics of the participants

	Males (*n* = 80)	Females (*n* = 76)	Total (*n* = 156)
Age (y)	19.9 ± 1.0	19.8 ± 1.1	19.8 ± 1.1
Height (cm)	172.0 ± 5.5	160.1 ± 6.1	166.2 ± 8.3
Weight (kg)	67.5 ± 11.9	54.8 ± 6.6	61.3 ± 11.5
BMI (kg/m^2^)	22.8 ± 3.9	21.4 ± 2.2	22.1 ± 3.3

### Temperature and humidity

The average indoors and outdoors temperature for the 7 days was 21.8 °C ± 1.2 °C and 20.7 °C ± 2.4 °C, respectively, while the average indoor and outdoor humidity was 40% ± 7 and 36% ± 5%, respectively (Table 2). The average time that the participants spent outdoors and indoors was 4 h and 20 h for workdays, and 8 h and 16 h weekend days, respectively.

**Table 2:** Indoors and outdoors temperature and humidity for the seven days

	Indoors		Outdoors	
	Temperature (°C)	Humidity (%)	Temperature (°C)	Humidity (%)
Sunday	19.9	43	18.1	37
Monday	23.0	48	22.4	41
Tuesday	23.3	31	24.0	29
Wednesday	21.5	48	17.9	42
Thursday	21.5	40	21.0	36
Friday	22.2	35	19.2	35
Saturday	21.2	34	22.6	31
Averages	21.8 ± 1.2	40 ± 7	20.7 ± 2.4	36 ± 5

### Intracellular, extracellular fluid and fluid intake of the participants

The mean TBW for all participants was 32.8[28.0,39.2] kg, and the percentage of TBW/BW was 55.2 ± 6.2%. The TBW and percentage of TBW/BW for males was higher than those for females (*Z* = -10.570, *p* < 0.001; *t* = 9.750, *p* < 0.001). The mean ICF and percentage of ICF/BW were 20.5[17.3,24.5] kg and 34.4 ± 4.0%, respectively; both were higher in males than females (*Z* = − 10.611, *p* < 0.001; *t* = 10.342, *p* < 0.001). The mean ECF and percentage of ECF/BW were 12.4[10.7,14.7] kg and 20.8 ± 2.3%, respectively; both of which were higher in males than that of females (*Z* = − 10.408, *p* < 0.001; *t* = 7.977, *p* < 0.001). The percentage of ECF/ICF was 60.5 ± 1.7%, and no statistical significance between sexes was found.

The mean volume of TWI for all participants was 2373 ± 648 mL, and it was higher in males than that of females (*t* = 4.392, *p* < 0.001). The mean volume of TFI was 1135[858,1478] mL, and the percentage of TFI/TWI was 49.1 ± 9.5%. The TFI of males was higher than that of females (*Z* = -3.131, *p* = 0.002), there was no statistical significance in the percentage of TFI/TWI between sexes (*t* = 0.301, *p* = 0.764). The mean volume of FWI and percentage of FWI/TWI were 1173 ± 263 mL and 50.9 ± 9.5%, respectively. There was statistical significance in the FWI between sexes, which is higher among males than females (*t* = 6.054, *p* < 0.001), but no difference was found in FWI/TWI (*t* = 0.301, *p* = 0.764) (Table 3).

**Table 3:** The intracellular, extracellular fluid and fluid intake of the participants

	Males	Females	Total	Statistical value (t/Z)	*p*
ICF					
Content (kg)	24.5 [22.8,26.4]	17.3 [16.1,18.5]	20.5 [17.3,24.5]	-10.611^a^	< 0.001
ICF/BW(%)	36.9 ± 3.6	31.8 ± 2.3	34.4 ± 4.0	10.342^b^	< 0.001
ECF					
Content (kg)	14.7 [13.5,15.6]	10.6 [9.9,11.4]	12.4 [10.7,14.7]	-10.408^a^	< 0.001
ECF/BW (%)	22.0 ± 2.2	19.5 ± 1.5	20.8 ± 2.3	7.977^b^	< 0.001
TBW					
Content (kg)	39.1 [36.2,42.2]	28.0 [26.0,29.9]	32.8 [28.0,39.2]	−10.570_a_	< 0.001
TBW/BW (%)	58.8 ± 5.8	51.3 ± 3.8	55.2 ± 6.2	9.750^b^	< 0.001
ECF/ICF (%)	60.4 ± 1.7	60.6 ± 1.7	60.5 ± 1.7	−0.746^b^	0.475
TFI (mL)	1214 [977,1545]	958 [696,1383]	1135 [858,1478]	−3.131_a_	0.002
TFI/TWI (%)	49.3 ± 8.6	48.8 ± 10.4	49.1 ± 9.5	0.301^b^	0.764
FWI (mL)	1286 ± 263	1054 ± 213	1173 ± 263	6.054^b^	< 0.001
FWI/TWI (%)	50.7 ± 8.6	51.2 ± 10.4	50.9 ± 9.5	−0.301^b^	0.764
TWI (mL)	2583 ± 602	2154 ± 625	2373 ± 648	4.392^b^	< 0.001

Note: *ICF* Intracellular fluid, *ECF* Extracellular fluid, *TBW* Total body water, *TFI* Daily total fluid intake, *FWI* Daily water intake from food, *TWI* Daily total water intake. Mean and standard deviation (SD) was used to describe variables in normal distribution; percentile (P50 [P25, P75]) was used to describe variables in skewness distribution. ^a^, means the statistical value *Z* of Kruskal-Wallis test for comparison of indexes between sexes. ^b^, means the statistical value *t* of Student’s t-test for comparison of indexes between sexes

### Comparisons on distribution of body water among participants with different water intake

When compared with the recommendation for the adequate water intake (AI) for TFI set by *Chinese Nutrition Society (2013)* (adult male: 1700 mL; adult female: 1500 mL)*,* only 29 participants (18.6%) achieved AI for TFI; 15 males (18.8%) and 14 females (18.4%). The percentages of TFI achieving AI among male and female participant has no difference (χ2 = 0.003, *p* = 0.958). For males whose TFI met or exceeded the AI, the values of ICF, ECF and TBW were higher than those with TFI less than AI (*Z* = -1.985, *p* = 0.047; *Z* = -2.134, *p* = 0.033; *Z* = -2.053, *p* = 0.040) (Table 4).

**Table 4:** The differences of the contents of intracellular and extracellular fluid between participants whose TFI achieve AI^a^ or not

		N (%)	ICF (kg)	ICF/BW (%)	ECF (kg)	ECF/BW (%)	TBW (kg)	TBW/BW (%)	ECF/ICF (%)
Total	<AI	127 (81.4%)	20.3 [17.6,24.4]	34.1 ± 4.0	12.3 [10.7,14.4]	20.6 ± 2.3	32.7 [28.3,38.4]	54.8 ± 6.3	60.4 ± 16.8
	≥AI	29 (18.6%)	23.2 [17.2,25.5]	35.4 ± 3.5	13.6 [10.6,15.1]	21.4 ± 2.2	37.0 [27.8,40.4]	56.9 ± 5.7	61.0 ± 16.7
	Statistical value (t/Z/χ^2^)		−0.863^b^	−1.566^c^	− 0.945^b^	− 1.717^c^	− 0.884^b^	−1.632^c^	− 1.663^c^
	*p*		0.388	0.119	0.344	0.088	0.377	0.105	0.098
Males	<AI	65 (81.3%)	24.0 [22.2,26.3]	37.1 [34.3,39.2]	14.4 [13.4,15.5]	22.0 [20.6,23.1]	38.3 [35.7,41.9]	54.8 ± 6.3	60.4 ± 16.8
	≥AI	15 (18.8%)	24.8 [23.9,26.9]	38.8 [35.9,40.5]	15.1 [14.6,16.1]	22.9 [21.6,24.2]	39.9 [38.7,43.4]	56.9 ± 5.7	61.0 ± 16.7
	Statistical value (t/Z/χ^2^)	0.003 ^d^	−1.985^b^	−1.570^b^	−2.134^b^	−1.447^b^	−2.053^b^	−1.632^c^	− 1.663^c^
	*p*	0.958	0.047	0.116	0.033	0.148	0.040	0.105	0.098
Females	<AI	62 (81.6%)	17.5 [15.9,18.5]	31.6 [30.0,33.3]	10.6 [9.8,11.4]	19.3 [18.4,20.5]	28.2 [25.8,29.9]	50.8 ± 3.3	60.4 ± 17.1
	≥AI	14 (18.4%)	17.2 [16.4,18.4]	31.7 [30.6,34.0]	10.6 [10.1,11.1]	19.5 [18.6,20.6]	27.8 [26.8,29.5]	53.5 ± 5.0	61.3 ± 16.4
	Statistical value (t/Z/χ^2^)		−0.08^b^	−0.484^b^	−0.013^b^	−0.563^b^	−0.040^b^	−1.947^b^	−1.773^c^
	*p*		0.936	0.628	0.989	0.573	0.968	0.070	0.080

Note: *ICF* Intracellular fluid, *ECF* Extracellular fluid, *TBW* Total body water. ^a^; TFI was compared with the recommendation for the adequate water intake (AI) for TFI set by Chinese Nutrition Society (2013) (adult male: 1700 mL; adult female: 1500 mL). Mean and standard deviation (SD) was used to describe variables in normal distribution; percentile (P50 [P25, P75]) was used to describe variables in skewness distribution; and count data were presented as n (percentage). ^b^, means the statistical value *Z* of Kruskal-Wallis test for comparison of indexes between sexes. ^c^, means the statistical value *t* of Student’s t-test for comparison of indexes between sexes. ^d^, means the statistical value *χ2* of *χ2* test for comparison of indexes between sexes

Only 31 participants (19.9%) (17 males, 21.3% and 14 females, 18.4%) achieved the AI for TWI (adult male: 3000 mL; adult female: 2700 mL). The percentages of TFI achieving AI among male and female participant has no difference (χ2 = 0.196, *p* = 0.658). The values of ECF/BW and TBW/BW among participants whose TWI met or exceeded the AI were higher than those with TWI less than AI (*t* = − 2.165, *p* = 0.036; *t* = − 2.011, *p* = 0.046). Among both males and females, the values of TBW/BW in participants whose TWI met or exceeded the AI were higher than those with TWI less than AI (*t* = − 2.011, *p* = 0.046; *t* = − 2.716, *p* = 0.008) (Table 5).

**Table 5:** The differences of the contents of intracellular and extracellular fluid between participants whose TWI achieve AI^a^ or not

		N (%)	ICF (kg)	ICF/BW(%)	ECF (kg)	ECF/BW(%)	TBW (kg)	TBW/BW(%)	ECF/ICF (%)
Total	<AI	125 (80.1%)	20.3 [17.3,24.4]	34.1 ± 4.0	12.2 [10.5,14.5]	20.6 ± 2.2	32.7 [27.7,38.5]	54.7 ± 6.2	60.5 ± 17.3
	≥AI	31 (19.9%)	23.2 [17.4,24.8]	35.6 ± 3.8	13.6 [10.8,14.9]	21.6 ± 2.3	37.0 [28.2,39.9]	57.2 ± 6.1	60.6 ± 15.6
	Statistical value (t/Z/χ^2^)		−1.086^b^	−1.884^c^	−1.566^c^	−2.165^c^	−1.566^c^	−2.011^c^	−0.255^c^
	*p*		0.277	0.061	0.119	0.036	0.119	0.046	0.799
Males	<AI	63 (78.8%)	24.3 [22.2,26.3]	37.1 [34.6,39.2]	14.4 [13.4,15.6]	22.1 [20.7,23.1]	38.4 [35.7,42.0]	54.7 ± 6.2	60.5 ± 17.3
	≥AI	17 (21.3%)	24.6 [23.7,26.7]	38.8 [34.9,40.5]	14.8 [14.2,16.0]	22.7 [20.8,24.2]	39.6 [37.8,42.7]	57.2 ± 6.1	60.6 ± 15.6
	Statistical value (t/Z/χ^2^) 0.196 ^d^		−1.100^b^	−1.229^b^	−1.365^b^	−0.865^b^	−1.188^b^	−2.011^b^	−0.255^c^
	*P* 0.658		0.271	0.219	0.172	0.387	0.235	0.046	0.799
Females	<AI	62 (81.6%)	17.3 [15.9,18.4]	31.7 [30.3,33.3]	10.5 [9.8,11.4]	19.3 [18.5,20.6]	27.7 [25.8,29.9]	50.8 ± 3.4	60.6 ± 17.6
	≥AI	14 (18.4%)	17.4 [16.4,19.3]	31.3 [30.3,32.1]	10.8 [10.1,11.5]	19.2 [18.5,23.4]	28.1 [26.8,30.8]	53.7 ± 4.6	60.7 ± 16.0
	Statistical value (t/Z/χ^2^)		−0.784^b^	−0.697^b^	−0.751^b^	−0.409^b^	− 0.771^b^	−2.716^b^	−0.217^b^
	*p*		0.433	0.486	0.453	0.683	0.441	0.008	0.829

Note: *ICF* Intracellular fluid, *ECF* Extracellular fluid, *TBW* Total body water. ^a^; TWI was compared with the recommendation for the adequate water intake (AI) for TWI set by Chinese Nutrition Society (2013) (adult male: 3000 mL; adult female: 2700 mL). Mean and standard deviation (SD) was used to describe variables in normal distribution; percentile (P50 [P25, P75]) was used to describe variables in skewness distribution; and count data were presented as n (percentage). ^b^, means the statistical value *Z* of Kruskal-Wallis test for comparison of indexes between sexes. ^c^, means the statistical value *t* of Student’s t-test for comparison of indexes between sexes. ^d^, means the statistical value *χ2* of *χ2* test for comparison of indexes between sexes

### Correlation between intracellular, external fluid and fluid intake

Among all participants, there was moderate correlation between water intakes (TFI/BW, FWI/BW and TWI/BW) and body fluid (ICF/BW,ECF/BW and TBW/BW) (*p* < 0.01 for all), which was shown in Table 6. Same correlations were found among both males and females (Table 6).

**Table 6:** Correlation between intracellular, external fluid and fluid intake

		ICF/BW (%)		ECF/BW (%)		TBW/BW (%)		ECF/ICF (%)	
		*r*	*p*	*r*	*p*	*r*	*p*	*r*	*p*
Total	TFI/BW (mL/kg)	0.324^b^	< 0.001	0.324^b^	0.001	0.323^b^	< 0.001	−0.041^b^	0.608
	FWI/BW (mL/kg)	0.444^a^	< 0.001	0.470^a^	< 0.001	0.466^a^	< 0.001	0.119^a^	0.139
	TWI/BW (mL/kg)	0.402^b^	< 0.001	0.427^b^	< 0.001	0.410^b^	< 0.001	0.029^b^	0.720
Males	TFI/BW (mL/kg)	0.348^b^	0.002	0.329^b^	0.003	0.341^b^	0.002	0.006^b^	0.959
	FWI/BW (mL/kg)	0.567^a^	< 0.001	0.590^b^	< 0.001	0.581^a^	< 0.001	0.200^a^	0.075
	TWI/BW (mL/kg)	0.511^b^	< 0.001	0.509^b^	< 0.001	0.509^b^	< 0.001	0.085^b^	0.456
Females	TFI/BW (mL/kg)	0.407^b^	< 0.001	0.353^b^	0.002	0.383^b^	0.001	−0.074^b^	0.524
	FWI/BW (mL/kg)	0.576^a^	< 0.001	0.496^b^	< 0.001	0.577^a^	< 0.001	0.092^a^	0.428
	TWI/BW (mL/kg)	0.486^b^	< 0.001	0.446^b^	< 0.001	0.468^b^	< 0.001	0.000^b^	0.999

Note: *ICF* Intracellular fluid, *ECF* Extracellular fluid, *TBW* Total body water, *TFI* Daily total fluid intake, *FWI* Daily water intake from food, *TWI* Daily total water intake. ^a^, means the correlation coefficient *r* of *Pearson*. ^b^, means the correlation coefficient *r* of *Spearman*

### Multiple linear regression model on water intake and body fluid

Multiple Linear Regression Model on ICF/BW was developed using gender, TFI/BW, FWI/BW and TWI/BW as independent variables. The percentage of variance in ICF/BW (R^2^) explained by the model was 78.6%, with a root mean square error of 2.5. In the model, gender, FWI/BW and TWI/BW were identified as possible key predictors of ICF/BW. Independent variable TFI/BW was excluded for the reason that there was no statistical significance. Same method of developing models on ECF/BW, TBW/BW and ECF/ICF were performed (Table 7). The percentage of variance in ECF/BW, TBW/BW and ECF/ICF (R^2^) explained by the model was 74.4, 77.4 and 54.5%, respectively; with root mean square errors of 1.5, 4.0 and 1.4, respectively.

**Table 7:** Multiple Linear Regression Model on water intake and body fluid

	Constant	Gender		FWI/BW		TWI/BW	
		ß	*p*	ß	*p*	ß	*p*
ICF/BW	24.497	5.092	< 0.001	0.242	< 0.001	0.065	0.007
ECF/BW	14.817	2.478	< 0.001	0.158	< 0.001	0.04	0.006
TBW/BW	39.285	7.556	< 0.001	0.402	< 0.001	0.105	0.006
ECF/ICF	60.549	−1.789	< 0.001	0.044	0.197	0.000	0.994

## Discussion

For young adults in this study, the values of TBW, ICF and ECF were 32.8 kg, 20.5 kg and 12.4 kg, which accounted for 55.2, 34.4 and 20.8% of the BW. There were sex differences in all these values with males being higher than females. The percentages of ECF/ICF was 60.5%, there was no sex difference. Some studies has demonstrated that TBW varies with different age, sex and ethnicity. TBW ranges from 38 to 46 L in white men, 26 to 33 L in white women [32]. In Korea, the TBW among male age < 30 years old was 38.5 L (accounting for 57.4% of body weight) and female 27.5 L (accounting for 52.3% of body weight) [33]. In the study of Chumlea 2001, it was found that the TBW among male (including white men and black men) age < 30 years old was 45.6 L and female 32.0 L [32]. Studies have been conducted in some disease states including kidney and liver disease (references) and another study has been conducted in male athletes in China [15, 16, 20–24]. It was found that the percentages of body weight for ICF, ECF and TBW among basketball players were 43.3, 21.4 and 64.7%, respectively; 41.9, 20.0 and 61.9% for football player; and 44.4, 21.5 and 65.8% for javelin throwers [34]. These data provides useful information for developing reference value of body fluid for population.

The volume of TWI, TFI and FWI were 2373 mL, 1135 mL, and 1173 mL among young adults in this study, respectively; and there was sex differences: male higher than female. The contribution of TFI to TWI was 49.1%. Few surveys of water intake have been conducted in China. In one survey among 1483 adults in China, TWI, TFI and FWI were 3045 mL, 1488 mL, and 1157 mL, respectively; TFI of males were higher than females; and the contribution of TFI to TWI was 55.8% [35]. The differences of the two study may be due to the differences in age range (18–23 years old vs 18–60 years old) and season (end of winter and the beginning of spring vs. summer). Another study among 1584 participants aged 18–55 years old found that the TFI was 1387 mL. Another study conducted among 68 young male adults with same age range and season, and it was found that the TWI, TFI and FWI were 2553 mL, 1342 mL, and 1211 mL, respectively; which has the similar results to the present study [19]. Compared to other countries, in a systematic review including 273 studies, it was concluded that the TWI was 0.8–3.4 mL/day among adults [36]. The data obtained from these water intake surveys provides useful information for developing recommendations on water AI for populations. Water intake varied among participants with age, sex, physical activity levels and environments. Thus, more water intake surveys among population with different age, sex, physiological stage and occupations in different environment should be conducted to get more data.

Water intake participates in the regulation of the balance of water metabolism. Distribution of body fluid is affected by the water balance. In addition, some studies showed that both water intake and body fluid had implications for healthy hydration. Is it possible that water intake perturb the distribution of water between ECF and ICF? Currently, no studies have been conducted to analyze the differences of the contents of intracellular and extracellular fluid between participants whose TFI or TWI achieve AI or not. In this study, it was the first time to explore the differences of body fluid among adults after grouping according to their water intake achieving corresponding AI or not. Although there was no difference in most of the body fluid indicators, some differences on body fluid were found after grouping. However, the difference was very small with larger *P* value which might be from a chance. The findings in this study may just have implications for that whether TFI or TWI achieve AI or not may do not disturb the balance on the distribution of body fluid obviously. In one study with rats, it was found that there is association between fluid intake and body fluid: acute absolute body-fluid deficits induced by injection of the diuretic drug furosemide in rats could cause the reduction of ECF and ICF value up to 20 and 2% respectively; and ICF was apparently restored after drinking [37]. Due to the limited extrapolation of animal experimental results, the effect of fluid intake on body fluid among population is still needed to be verified. In the study of Kazumasa et al. with 34 male elders, it was found that nocturnal urine volume was correlated with the difference in ECF at 8 a.m. and 9 p.m.. Urination is the main way of discharging fluid, and has a certain relationship with body fluid [38]. Then, are the main sources of water: TFI and TWI correlated with body fluid? In this study, water intakes (TFI/BW, FWI/BW and TWI/BW) were moderately correlated with body fluid (ICF/BW,ECF/BW and TBW/BW) among female participants, but not among male participants. In the study of Patterson et al., during 17 days of controlled hyperthermia, progressive dehydration state was induced among eight male participants, it was found that during progressive dehydration, plasma reduced of 9.0% (day 1), 12.4% (day 8) and 13.6% (day 22) and during recovery, plasma volume commenced to restore, with the contribution of ICF becoming more pronounced as acclimation progressed [39]. No other studies related water intake and body fluid have been reported. Body water homeostasis, expressed as fluid balance, is an indicator of health [40]. Body fluid balance is determined by water intake and water output. One study with 458 American adults 40–79 years old demonstrated that water turnover is highly variable among individual and that little of the variance is explained by anthropometric indexes [41]. Although the association between fluid intake and body fluid has been founded, more studies are needed in healthy people. And, further studies should be conducted to find the diagnostic threshold on TFI and TWI which may disrupt the distribution of body fluid, and it will be of significance for prevent some diseases related to water intake and body fluid, such as diseases of urinary system.

It is important to acknowledge the strengths and weaknesses in the present study. In terms of strengths, this was the first time to study the association of fluid intake and body fluid. The method of assessing water intake and body fluid are internationally recognized and authoritative. Referring to the methods of assessing water intake, the commonly used methods include 1 or 2 or 5 -day 24-h dietary recall, food frequency questionnaire, 3-day or 7-day 24-h fluid intake diary, weighing method and duplicate portion method, and so on. In the American National Health and Nutrition Examination Survey (NHANES) 2005–2010, information on water intake was collected by two non-consecutive day 24-h dietary recalls method [42]. In 2010, Veitch et al. adopt quantitative food frequency questionnaire to obtain estimates of water intake among 747 adolescents in Netherlands [43]. In China, in water intakes surveys for adults and children, 7-day 24-h fluid intake diary was used to assess TFI, weighing method and duplicate portion method for 3 consecutive days was adopted to assess FWI [17, 19, 44, 45]. The 7-day 24-h fluid intake diary is the only validated methodology for assessing TFI [46], and weighing and duplicate portion method for assessing FWI [26, 47]. Referring to the methods of assessing body fluid, bioelectrical impedance spectroscopy, bioelectrical impedance analysis, trace quantities of deuterium oxide and ultrasound are commonly used [28, 48–51]. It was widely recognized that human body composition analyzer based on bioelectrical impedance theory could be used to record body fluid distribution with high accuracy [52, 53]. The survey was conducted in free-living conditions, which may make the results more consistent with the nature and actual situation in life. In terms of weaknesses, this was a short term study and longer studies are needed. It is necessary to conduct further study to lengthen the time of dietary measurement and body fluid composition analysis. It does not take into account the possible differences between water intake and intracellular and extracellular fluids in North-South regions or other aspects of human body. The mechanism about the correlation between water intake and body fluid distribution was not explored.

## Conclusions

In conclusion, there is a certain degree of association between water intake and body fluid. Water AI is not a value to judge whether the body fluid balance is disturbed or not. More studies are needed to conducted to obtain scientific data on water intake and body fluid among different population. These studies will provide data for developing reference value on water intake and body fluid among population. And these will be helpful to find meaningful diagnostic threshold on TFI and TWI to prevent related diseases. It is also necessary to carry out fluid intake related health education to promote enough water intake based on scientific evidences of these related studies.

## Data Availability

The datasets used during the current study are available from the corresponding author on reasonable request.
